# Which young people in England are most at risk of an alcohol-related revolving-door readmission career?

**DOI:** 10.1186/s12889-016-3891-2

**Published:** 2017-02-11

**Authors:** Andrew R. Hoy

**Affiliations:** 1 National Institute for Health and Care Excellence (NICE), London, UK; 2 Ministry of Business, Innovation and Employment (MBIE), Wellington, New Zealand

## Abstract

**Background:**

This research investigated what Hospital Episode Statistics (HES) records could reveal about the development of problematic drinking careers among young people in England.

**Methods:**

A cohort of 7286 young people (aged 12-18) who had an index alcohol-related emergency admission between April 2003 and March 2004 were investigated for subsequent alcohol-related readmission. Regressions of patient and visit characteristics were performed against measures of readmission.

**Results:**

A total of 677 patients (9.3% of the cohort) were readmitted during the following 3.75 years, and this group had on average 1.52 readmissions following their index admission. Predictors of having a first readmission included living in a deprived area at index admission (B = -.081, OR = .923, 95% CI = .894 to .952, df = 1, *p* < .001); having another substance use diagnosis (B = .302, OR = 1.352, 95% CI = 1.017 to 1.798, df = 1, p < .05), or a comorbid mental health diagnosis (B = .441, OR = 1.555, 95% CI = 1.147 to 2.108, df = 1, *p* < .01), or a diagnosis of self-harm (B = .316, OR = 1.371, 95% CI = 1.082 to 1.738, df = 1, *p* < .01) at index admission. These last three results were also associated with the readmission rate being higher for young women than young men (B = -.250, OR = .779, 95% CI = .656 to .925, df = 1, *p* < .01). Patients who had an injury diagnosis alongside their alcohol diagnosis were less likely to be readmitted in the future (B = -.439, OR = .645, 95% CI = .475 to .876, df = 1, *p* < .01) On average, each subsequent admission featured a longer hospital stay; was progressively more likely to occur on a non-traditional drinking day; and occurred after a progressively smaller number of days since previous admission.

**Conclusions:**

This study illustrates ways in which problematic drinking careers can be analysed using routinely collected health information, and the results from this analysis may be useful in informing the process of hospital screening and treatment referral. The effects of poverty and comorbid conditions on the initiation of a drinking career are suggested by these results.

## Background

Recent reported falls in binge drinking among young people in Great Britain [1] are to be welcomed, but the level of binge drinking among 15-16 year olds still compares unfavorably with levels in other European countries, and alcohol use in general still costs the United Kingdom £21 billion a year [2]. One study in a British hospital found that alcohol was implicated in 21% of admissions to the Emergency Department (ED), or the Accident and Emergency (A&E) Department as they are called in Great Britain [3].

Alcohol use is cited as being associated with a generally higher rate of hospital readmission [4, 5]. In an audit of alcohol-related admissions in Leicestershire, 146 frequently attending patients with alcohol-related problems were identified (defined as having three or more admissions) and they attended hospital 647 times, at an estimated cost of £632,753 [6].

What differentiates those young people who will only ever have a single alcohol-related hospital admission (perhaps just as the result of inexperience with alcohol) from other young people who go onto develop a problematic drinking “career”? And if such an analysis was able to single out those who were most likely to persist with a heavy drinking career, could the results tell us how to detect potentially problematic drinkers before this career properly got underway [7]?

Are there clinical indicators which would enable clinicians to move from a situation of universal screening of such admissions to selective enquiry, therefore saving resources? There is a clear relationship between problematic drinking by adolescents and young people, and problematic drinking in later life [8–10]. Screening and early intervention therefore saves money in the longer term, by preventing further alcohol-related hospital admissions. In recognition of this, the National Institute for Health and Care Excellence’s (NICE) 2010 guidance on the prevention of alcohol-related problems [11] recommended routine screening and early intervention, where appropriate.

The aim of the present analysis was to gain a clear picture of the dynamics of alcohol-related readmissions among a cohort of young people in England, using National Health Service (NHS) Hospital Episode Statistics (HES) records for England [12]. The readmissions of a cohort of young people with alcohol-related admissions were followed over subsequent years. The focus of this research therefore was on the readmission of a cohort with alcohol-related admissions from the general population back into non-specialist hospital services. By focusing on admissions among the general population, the expectation was that the readmission dynamics observed would provider a wider view than the study of just those in specialist alcohol treatment. The majority of heavy drinkers are not in treatment, and there are difficulties generalising existing longitudinal outcome research on people in clinical treatment to this wider group [13].

Little previous research has looked at the readmission dynamics of untreated populations, especially young untreated drinkers. Previous research has tended to focus on cohorts in treatment already identified as having an alcohol problem, and their readmission rates back into specialist alcohol treatment [14, 15], or has focused on more general outcome measures like psychosocial outcomes [7]. Other studies have looked at readmission into general hospital services, but were studies centred around cohorts identified through treatment programmes, or studies centred around the intervening effect of treatment [16–19]. Only one Swedish study, which included patients from Stockholm county, has looked at readmission rates among a cohort both recruited in general hospital services, and readmitted to general hospital services, although this involved a much older cohort (mean age of 43.5 years) than the present study [10].

In contrast to previous research, and in particular the Stockholm research, the present study restricts the analysis to a young cohort, and also examines hospital readmission within a nation as a whole. Unlike the Stockholm study, the present study also eliminates those with a prior history of problematic alcohol use from the cohort, allowing readmission dynamics to be analysed without the confounding effects of patient history prior to the study period. The present study also undertook an alternative way of recording and analysing the often multiple readmissions of the cohort members, storing them in table in a sequence and analysing their characteristics on that basis. Finally this study uses additional independent variables to study the characteristics of readmission, perhaps the most important addition being a measure of imputed patient socioeconomic background.

## Methods

The present study was a retrospective cohort study design. In summary: HES records for England were searched to find follow-up readmission records of a cohort of young people (aged 12-18) who were formally admitted (usually, but not always, overnight) as an alcohol-related emergency patient (as opposed to an electively admitted patient) between April 2003 and March 2004. (Note that this definition of admission excludes people seen in an ED and discharged relatively quickly.) Regression was then applied in an attempt to find predictors of future alcohol-related readmission.

### Sample selection

The sample frame of the cohort was all HES hospital records for England for emergency admission episodes that included specified alcohol diagnosis codes and which began at some point during the year of 1 April 2003 to 31 March 2004. HES records are an immensely powerful dataset with which to analyse alcohol problems, offering total coverage of both (non-private) hospital usage among the population of England, and their ED admissions.

**Fig. 1:**
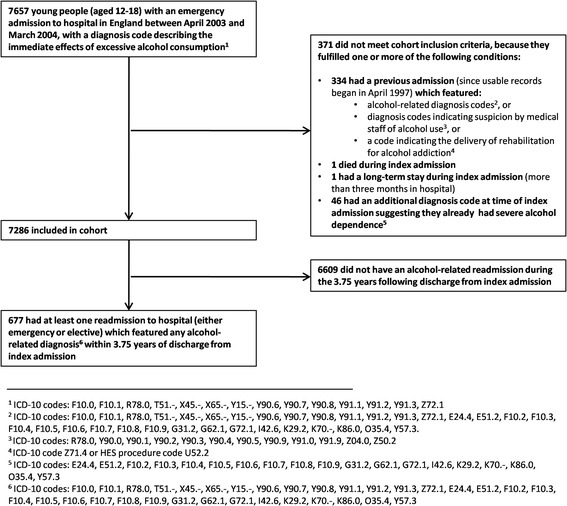
Patient flowchart

Figure 1 gives a patient flowchart. In summary, the young people who were selected were all intoxicated during an emergency admission to hospital (although alcohol may not have been the primary reason why they were admitted). As shown this diagram, inclusion or exclusion in the study was through careful selection, based on the International Classification of Diseases-10 (ICD-10) codes appearing on a patient's admission record. Bearing in mind that the aim of the research was to select a cohort of young people who were in danger of establishing a problematic drinking career, the exclusion of two groups of patients was seen as particularly important. First, it was decided to exclude the relatively small number of patients who appear to have developed severe alcohol dependence at a young age, on the basis that they seemed a distinct "type" of patient, separate from those in the spotlight of this research. Therefore patients were excluded from the sample if they had any one of a selected range of diagnoses (for example, alcoholic liver disease, Wernicke's encephalopathy, or degeneration of nervous system due to alcohol). This led to 46 young people being excluded from the cohort. Second, there was a group of 334 patients who were presenting (like all others in the cohort) with a code which only indicated intoxication rather than dependence, but for whom this was not their first alcohol-related admission (according to the six years of previous usable HES data). This group was also eliminated from the cohort.

Patients were also eliminated from the cohort if they died during this index admission in hospital, or if their index stay in hospital was 90 or more days in length, the latter criteria being adopted so that the follow-up period of the study could be as long as possible.

### Criterion variable – readmission

The first part of the analysis needed a simple binary variable to indicate whether patients had been readmitted or not. Emergency or elective alcohol-related admissions to hospital for both the cohort study period of the 2003/04 financial year, and the four financial years following (up to 31 March 2008) were extracted and placed in a separate “follow-up” dataset. This dataset was matched to the people involved in the index admissions, through the use of the anonymised patient identification number available on the version of HES records available to researchers. The presence of any patient identification code matches (a “readmission”) resulted in a "1" being recorded on the readmission indicator. To standardise this variable across all patients, only admissions that occurred up to 3.75 years (1370 days) after the patients’ index discharges were counted, to allow for the fact that patients could be admitted on the last day of the selection period and then could spend up to three months in hospital, while still remaining eligible for the cohort. Unlike the codes used to select the cohort originally, which excluded any admissions with diagnosis codes implying a severe alcohol dependence, no such restriction was applied to the follow-up cohort. Therefore an alcohol-related readmission was recorded as having happened for a patient if they had any of the codes listed at the bottom of Fig. 1.

For two other regressions performed, two additional criterion variables were derived from this database for those patients who had at least one alcohol readmission. These were two continuous variables which counted, respectively, the total number of alcohol-related readmissions each patient had during the 3.75 years; and the number of days between their index admission and their first readmission. Both of these variables are attempts to measure how serious an alcohol problem is for a young person who has started to manifest multiple readmissions.

### Predictor variables for the regressions

Because this was intended as an initial exploratory study, a wide range of other predictor variables (see Table 1) was calculated from the HES records and other data sources, and tested as predictors of readmission. Most of the predictor variables were chosen for the regression on the basis of previous published research, or because they related to accompanying diagnoses that are common in alcohol-related admissions in England. Other variables were included on the basis of a prior demonstrated bivariate correlation with readmission by the present author, correlations given in Table 1.

**Table 1:** Clinical, demographic, and visit characteristic predictor variables for the regression

Variable	Method of derivation	Bivariate correlation (Kendall’s Tau) with any alcohol re-admission	Investigated or proposed in previous literature
**Diagnosis variables**			
Patient also had self-harm diagnosis at time of index episode (0 = no, 1 = yes)	Did the patient have an additional ICD-10 code of X60 to X84, but excluding X65	.110***	
Patient also had assault diagnosis at time of index episode (0 = no, 1 = yes)	Did the patient have an additional ICD-10 code of X85 to X99, Y00 to Y09, or T741?	.017	[21]
Patient also had “undetermined intent” diagnosis at time of index episode (0 = no, 1 = yes)	Did the patient have an additional ICD-10 code of X60 to X84, or Y10 to Y34? If the patient had an ambiguous coding – i.e., an “undetermined intent” code mixed with a self-harm or an assault diagnosis, then it was coded as being undetermined intent only. Also if the patient had a mixed assault and self-harm diagnosis, they were also coded as undetermined intent only.	-.002	
Patient also had injury diagnosis at time of index episode (0 = no, 1 = yes)	Did the patient have an additional ICD-10 code of S*, T00-T32, T66, T67, T70, T71, T75, V*, W*, X00-X39, X50-X52? Excluded from this variable were cases where the patient also had another diagnosis which suggested that the injury was not a straightforward accident – i.e., a parallel coding of self-harm; assault; or event of undetermined intent.	-.042***	[21]
Patient also had other mental health diagnosis at time of index episode (0 = no, 1 = yes)	Did the patient have an additional ICD-10 code of F0 to F9, but excluding F7?	.061***	[7, 10, 16, 17]
Patient also had other substance use diagnosis at time of index episode (0 = no, 1 = yes)	Did the patient have an additional ICD-10 code of F11 to F19, T40, or X42?	.053***	[7, 10, 16, 17]
Patient’s primary diagnosis at index admission was alcohol related (0 = no, 1 = yes)	Was the first (primary) diagnosis recorded in HES for this admission an alcohol related code, or was this recorded as a secondary code?	-.087***	[20]
**Demographic variables**			
Average level of wealth of the patient’s area of residence at time of index admission (interval variable, ranging from 1 to 10)	Calculated from Index of Multiple Deprivation (IMD) coding of the patient’s address, and taken from the demographic fields of the HES index admission record	-.062***	[22]
Age at start of index admission (interval variable, ranging from 12-18 years)	Recorded on the index admission record as a demographic field	.032**	
Patient was treated during index admission in an urban Primary Care Trust (PCT) (0 = no, 1 = yes)	Based on a pre-existing methodology and codings [31] and the classifications of the Office of National Statistics, did the patient have their index admission in a hospital that was in an “urban” PCT area, as opposed to a PCT in a “rural” area?	.009	
Patient gender (0 = female, 1 = male)	Recorded on the index admission record as a demographic field	-.047***	[7, 10]
Address missing at time of index admission (0 = no, 1 = yes)	Was there a missing address at the time of the patient’s current admission – as indicated by a missing Local Authority code listed against their record?	-.003	
**Admission characteristic variables**			
Length of stay in hospital during the index admission (continuous variable, expressed in days)	Admission date subtracted from discharge data, expressed in days	.030**	
Patient had index admission on a “traditional” drinking day (0 = no, 1 = yes)	Did the patient have their index admission on a day that was: (a) a Friday, Saturday, or Sunday, (b) a public holiday, or (c) a day on the eve of a public holiday?	-.043***	[23, 24]
Outside ambient air temperature on date of index admission (continuous variable, expressed in degrees Celsius)	The mean daytime temperature on the date of admission according to the Hadley Centre Central England Temperature (HadCET) dataset [32]	.008	

* *p* < .05, ** *p* < .01, *** *p* < =.001 (two tailed)

The first group of predictors listed in Table 1 consists of diagnosis variables that reflect the types of other comorbid conditions that young people with alcohol-related emergency admissions also often have. The next group of predictor variables - demographic attributes - is mostly self-explanatory. The “address missing” variable was used because it was assumed that this was a possible indicator of homelessness. A final group of predictor variables measure hospital stay characteristics, and circumstances surrounding the index admission. Two of the three variables measure the extent to which the circumstances of the index admission date might have predisposed someone towards drinking, specifically indicating whether it was a “traditional” drinking day, and whether the weather was hot.

### Additional descriptor variables for sequence analysis of patients undergoing multiple readmissions

A secondary dataset was prepared that recorded patient and visit characteristics at each return visit, for those who were readmitted one or more times. A secondary part of the analysis – a breakdown of patient characteristics at each readmission in sequence – can give us a descriptive cross-section of an "average" problematic drinking career. Two additional variables calculated for this part of the analysis were: a binary measure of whether a patient died during a given admission episode (according to the HES “discharge destination” code for that record); and a measure of the length of time (in days) since a patient's last discharge from alcohol-related admission.

## Results

### Cohort composition

The total number of young people who were admitted for an emergency treatment between April 2003 and March 2004 who had an alcohol-related ICD-10 code as either their primary or secondary diagnosis code was 7286. Selected breakdowns of the cohort are listed in Table 2. Males made up 48.8% of the cohort, and 51.2% were female. There was no readmission related to alcohol use after index admission by 90.7%, but 6.9% were readmitted once for this reason, 1.6% of the cohort were readmitted twice, and 0.4% were readmitted three times. The extent of missing ethnicity data (42.5%), as evident in Table 2, shows why no useful analysis based on this attribute could be undertaken.

**Table 2:** Characteristics of the cohort

	Number	Percent
**Primary diagnosis code at time of index admission**		
Intoxication/Mental and behavioural disorders due to alcohol use (F10)	3608	49.5%
Poisoning by drugs, medicaments and biological substances (T36–T50)	1382	19.0%
Head Injury (S00–S09)	885	12.1%
Toxic effect of alcohol (T51)	579	7.9%
Other physical injury (S10-S99, T00-T35)	378	5.2%
Syncope and collapse (R55)	112	1.5%
Diseases of the digestive system (K00-K93)	50	0.7%
Digestive system and abdomen conditions (R10–R19)	43	0.6%
Convulsions (R56)	27	0.4%
Diabetes mellitus (E10–E14)	22	0.3%
Epilepsy (G40)	19	0.3%
Intoxication/Mental and behavioural disorders due to (non-alcoholic) psychoactive substance use (F11-F19)	16	0.2%
Other primary diagnosis	165	2.3%
**Demographics at time of index admission**		
Males	3553	48.8%
Females	3733	51.2%
White	3977	54.6%
Asian or Asian British	64	0.9%
Black or Black British	54	0.7%
Ethnicity mixed or other	96	1.3%
Ethnicity not known or not stated	3095	42.5%
Aged 12	332	4.6%
Aged 13	923	12.7%
Aged 14	1504	20.6%
Aged 15	1574	21.6%
Aged 16	925	12.7%
Aged 17	896	12.3%
Aged 18	1132	15.5%
**Comorbidity variables at time of index admission**		
Patient also had self-harm diagnosis	1515	20.8%
Patient also had injury diagnosis	1146	15.7%
Patient also had other substance use diagnosis	435	6.0%
Patient also had assault diagnosis	321	4.4%
Patient also had other mental health diagnosis	343	4.7%
Patient also had “undetermined intent” diagnosis	70	1.0%
**Number of alcohol-related readmissions following index admission**		
Didn't return after index episode	6609	90.7%
Returned only once (i.e., two visits in total)	501	6.9%
Returned only twice (three visits in total)	116	1.6%
Returned only three times (four visits in total)	29	0.4%
Returned only four times (five visits in total)	15	0.2%
Returned only five times (six visits in total)	5	0.1%
Returned six or more times (seven or more visits)	11	0.2%

(Baseline is 2003/04 emergency admissions, aged between 12 and 18, *n* = 7286)

**Fig. 2:**
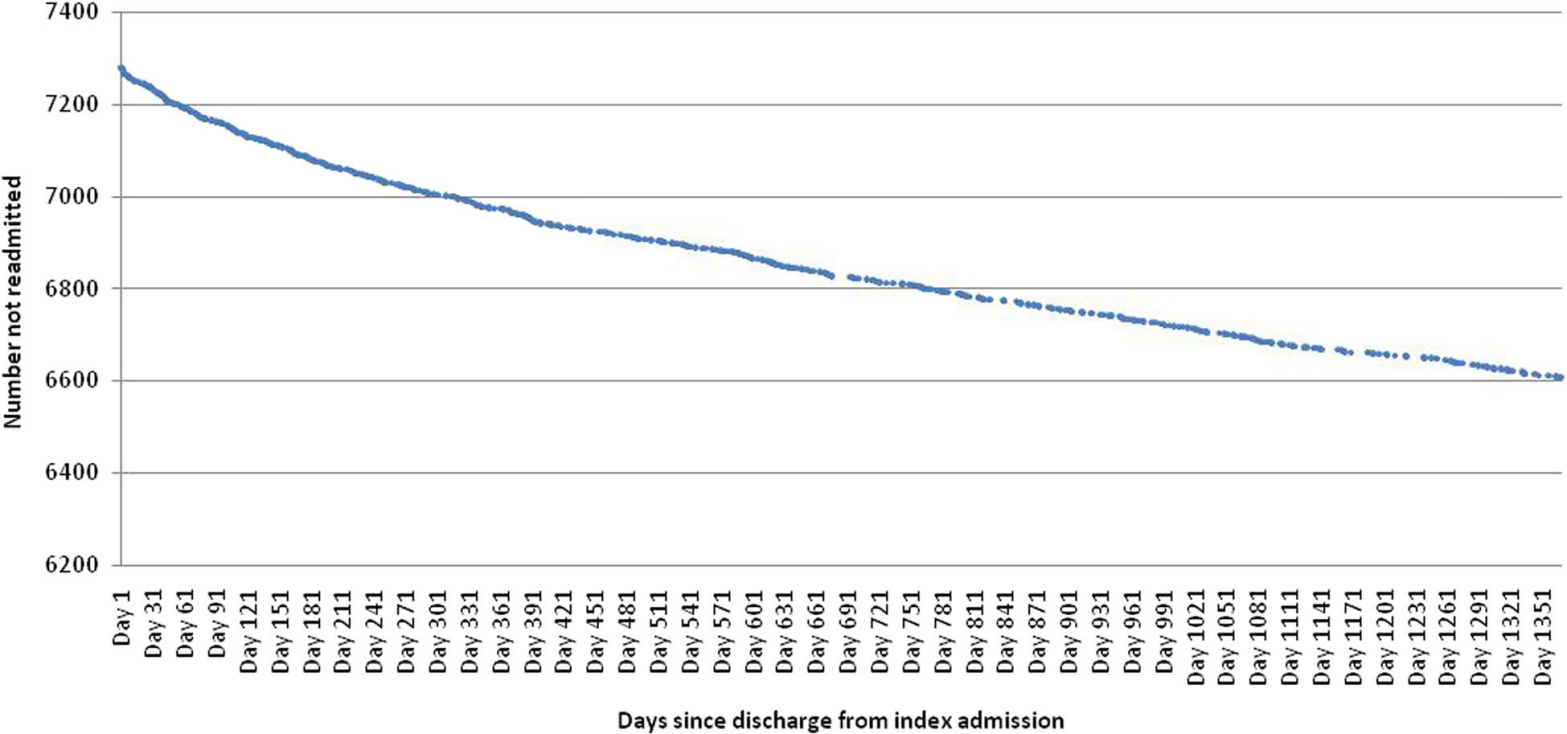
Survival curve of time before first alcohol-related readmission

A total of 677 patients (9.3% of the cohort) were readmitted during the following 3.75 years, and this group had on average 1.52 readmissions following their index admission. This ranged from one readmission (2 visits in total), to 25 readmissions (26 visits in total). Fig. 2 depicts a survival curve of the amount of time before first alcohol-related readmission among cohort members. This shows a steady concave decline in readmissions among the cohort. By day 151, 25% of the 677 young people who will be readmitted, have been readmitted, and by day 410, 50% of this group have been readmitted. The shape of the curve also strongly suggests that the readmission rate would have continued to cumulatively mount after the end of the 1370 day analysis period.

### Regression analysis of readmission

The third column of Table 1 features the bivariate correlation that each predictor variable had with the presence of any alcohol readmission among the whole cohort. Following the running of these initial correlations, regression analysis using all predictor variables together was conducted on the whole cohort (*n* = 7286) to predict readmission. In an attempt to shine a particular light on why some young people develop “revolving door” readmission careers, similar regressions were conducted on only the people with at least one readmission (*n* = 677) with respect to the other two criterion variables: the total number of times patients were readmitted in 3.75 years, and the number of days between discharge from their index admission and their first readmission.

Prior to analysis, the continuous predictor and criterion variables in the regressions were examined for distribution and outliers, and were deemed appropriate for use in regression analysis. Logistic regression was conducted for the first regression because the criterion variable was dichotomous, and linear regression was used for regressions two and three because the criterion variables were continuous. The method for all three regressions was forced entry. The Durbin-Watson statistic was also generated for the two linear regressions, and the figures were 2.081 and 2.026 respectively, suggesting that there was little evidence of positive serial correlation.

Multicollinearity was examined by calculating the average Variance Inflation Factor (VIF) across all 15 predictor variables, and this was low for the dataset used for both the full dataset used for the first regression (1.250), and the dataset used for regressions two and three (1.225). Secondly, the bivariate correlations between predictor variables were also examined, and there were no variable pairs that presented a cause for concern.

In the first regression, the binary logistic regression predicting any readmission (Table 3), the overall amount of variance explained was low (Cox & Snell R^2^ = .023). However, five diagnosis variables significantly predicted the likelihood of a young person coming back to hospital with another alcohol admission, the strongest being that when the patient’s primary diagnosis at index admission was alcohol related, they were significantly less likely to return than a patient whose secondary diagnosis was alcohol-related (B = -.489, OR = .613, 95% CI = .481 to .782, df = 1, *p* < .001). Other results suggest that return is significantly more likely to occur when a patient was admitted with during their index admission with: an additional mental health diagnosis (B = .441, OR = 1.555, 95% CI = 1.147 to 2.108, df = 1, *p* < .01); a diagnosis of self-harm (B = .316, OR = 1.371, 95% CI = 1.082 to 1.738, df = 1, *p* < .01); or an additional (i.e., non-alcohol) substance use diagnosis (B = .302, OR = 1.352, 95% CI = 1.017 to 1.798, df = 1, *p* < .05). On the other hand, an additional diagnosis-related finding from this table is that having a diagnosis of a physical injury (which did not result from self-harm or assault) at their index admission made a patient significantly less likely to come back for an alcohol readmission (B = -.439, OR = .645, 95% CI = .475 to .876, df = 1, *p* < .01).

**Table 3:** Binary logistic regression predicting any readmission (*n* = 7286)

	B	SE B	Wald's *X* ^2^	EXP(B) (odds ratios)	Lower 95% C.I. for EXP(B)	Upper 95% C.I. for EXP(B)	Sig
**Diagnosis variables**							
Patient also had assault diagnosis at time of index episode (0 = no, 1 = yes)	.197	.206	.915	1.217	.814	1.822	
Patient also had other substance use diagnosis at time of index episode (0 = no, 1 = yes)	.302	.145	4.315	1.352	1.017	1.798	*
Patient also had injury diagnosis at time of index episode (0 = no, 1 = yes)	-.439	.157	7.857	.645	.475	.876	**
Patient also had other mental health diagnosis at time of index episode (0 = no, 1 = yes)	.441	.155	8.070	1.555	1.147	2.108	**
Patient also had self-harm diagnosis at time of index episode (0 = no, 1 = yes)	.316	.121	6.817	1.371	1.082	1.738	**
Patient also had “undetermined intent” diagnosis at time of index episode (0 = no, 1 = yes)	-.030	.435	.005	.971	.413	2.279	
Patient’s primary diagnosis at index admission was alcohol related (0 = no, 1 = yes)	-.489	.124	15.479	.613	.481	.782	***
**Demographic variables**							
Average level of wealth of the patient’s area of residence at time of index admission	-.081	.016	25.513	.923	.894	.952	***
Age at start of index admission (within the 12-18 year age band)	-.022	.027	.613	.979	.927	1.033	
Patient was treated during index admission in an urban PCT (0 = no, 1 = yes)	-.079	.089	.798	.924	.776	1.099	
Patient was male (0 = no, 1 = yes)	-.250	.088	8.160	.779	.656	.925	**
Address missing at time of index admission (0 = not missing, 1 = missing)	.376	.317	1.409	1.456	.783	2.710	
**Admission characteristic variables**							
Length of stay in hospital during the index admission	.005	.022	.055	1.005	.962	1.050	
Patient had index admission on a “traditional” drinking day (0 = no, 1 = yes)	-.154	.087	3.109	.857	.722	1.017	
Outside ambient air temperature on date of index admission	.003	.008	.120	1.003	.988	1.018	
**Constant**	-1.190	.479	6.178	.304			*
** Tests**							
Omnibus Tests of Model Coefficients							
Step *X* ^2^ = 164.975,df = 15, *p* < .001							
Block *X* ^2^ = 164.975,df = 15, *p* < .001							
Model *X* ^2^ = 164.975,df = 15, *p* < .001							
Hosmer and Lemeshow Test *X* ^2^ = 17.311,df = 8, *p* = .027							
-2 Log likelihood 4331.445							
Cox & Snell R Square .023							
Nagelkerke R Square .049							

* *p* < .05, ** *p* < .01, *** *p* < =.001

Only two other variables in Table 3 significantly predict the likelihood of readmission. The variable recording the average level of wealth of the patient’s area of residence at time of index admission has a significant and negative value that (B = -0.081, OR = .923, 95% CI = .894 to .952, df = 1, *p* < .001), showing that greater imputed wealth was associated with less likelihood of alcohol-related readmission. The results for the variable noting male gender was also negative, suggesting that young men are less likely than young women to come back for a second alcohol-related readmission (B = -.250, OR = .779, 95% CI = .656 to .925, df = 1, *p* < .01).

Other predictor variables in the analysis failed to have a significant association with the criterion variable. The presence of a physical assault against a patient didn't have the same negative association with readmission that injury did, as might be expected, but the coefficient was not significant in any case. The patient age variable, and the variable indicating urban or rural location were other notable null-results. The “address missing” variable also didn’t produce results. It was noted above as being a possible indicator of homelessness, although it is acknowledged that this might not have been capturing situations where a patient is able to give a homeless shelter, for example, as their address.

The results were less marked for the regressions of the independent variables against the two other dependent variables recording the total number of times someone was readmitted, and the number of days between the discharge from their index admission and their first readmission (Table 4). The general lack of significant results may be because of the relatively small number of patient records (*n* = 677) in these two regressions. In the first of these two regressions, only a patient's length of stay in hospital during the index admission significantly predicts how many times they will return in total to hospital with an alcohol-related diagnosis (β = .153, df = 1, *p* < .001). Only three variables predict (in the second of the two regressions) the length of time between a patient's index admission and their first readmission, and shorter periods are significantly associated with the patient not having an “undetermined intent” diagnosis at time of index episode (β = .099, df = 1, *p* < .05); the patient being older (within the 12 to 18 year age band) at the time of the index admission (β = -.092, df = 1, *p* < .05); and the patient being female (β = .153, df = 1, *p* < .001). The total amount of variance explained overall in these two regressions was also small. For the total number of times readmitted, the R^2^ value was .025, and for the number of days since discharge from index admission, the R^2^ value was .034.

**Table 4:** OLS regressions predicting total number of readmissions, and number of days until readmission, among those with at least one readmission (*n* = 677)

	Regression against “total number of readmissions” variable (*n* = 677)				Regression against “number of days till first readmission” variable (*n* = 677)			
	B	SE B	*β*		B	SE B	*β*	
**Diagnosis variables**								
Patient also had assault diagnosis at time of index episode (0 = no, 1 = yes)	-.352	.302	-.049		-38.252	73.977	-.021	
Patient also had other substance use diagnosis at time of index episode (0 = no, 1 = yes)	-.164	.213	-.030		83.849	52.153	.062	
Patient also had injury diagnosis at time of index episode (0 = no, 1 = yes)	-.347	.233	-.066		-7.836	57.167	-.006	
Patient also had other mental health diagnosis at time of index episode (0 = no, 1 = yes)	.163	.229	.028		-71.524	56.274	-.050	
Patient also had self-harm diagnosis at time of index episode (0 = no, 1 = yes)	-.032	.175	-.009		-60.333	42.976	-.071	
Patient also had “undetermined intent” diagnosis at time of index episode (0 = no, 1 = yes)	-.534	.674	-.031		427.671	165.296	.099	*
Patient’s primary diagnosis at index admission was alcohol related (0 = no, 1 = yes)	-.207	.178	-.063		-65.637	43.610	-.081	
**Demographic variables**								
Average level of wealth of the patient’s area of residence at time of index admission	-.023	.025	-.037		5.199	6.087	.034	
Age at start of index admission (within the 12-18 year age band)	.053	.042	.057		-20.931	10.189	-.092	*
Patient was treated during index admission in an urban PCT (0 = no, 1 = yes)	-.027	.137	-.008		43.768	33.586	.052	
Patient was male (0 = no, 1 = yes)	-.072	.135	-.022		125.755	33.167	.153	***
Address missing at time of index admission (0 = not missing, 1 = missing)	.384	.477	.031		103.224	116.940	.034	
**Admission characteristic variables**								
Length of stay in hospital during the index admission	.208	.052	.153	***	-7.991	12.843	-.024	
Patient had index admission on a “traditional” drinking day (0 = no, 1 = yes)	-.159	.132	-.046		35.853	32.354	.043	
Outside ambient air temperature on date of index admission	-.002	.012	-.008		2.459	2.883	.033	
**Constant**								
	1.966	.731		**	728.453	179.320		***
	Model: 1				Model: 1			
	R: .216				R: .236			
	R Square: .047				R Square: .056			
	Adjusted R Square: .025				Adjusted R Square: .034			
	Std. Error of the Estimate: 1.622				Std. Error of the Estimate: 397.899			

* *p* < .05, ** *p* < .01, *** *p* < =.001

### Sequence analysis – characteristics of patients undergoing multiple readmissions

As we have seen, a small minority of cohort members established a revolving door drinking career, in that they had a second or subsequent readmission. Table 5 gives a breakdown of attribute by visit for all cohort members having up to five readmissions. The table shows that each subsequent visit is generally associated with: a longer average hospital stay; an increased tendency for the visit to be on a “non-traditional” drinking day; and for there to be a decreasing average time in days since their previous admission.

**Table 5:** Sequence analysis - patient and admission attributes by visit

	Stay characteristics of all those at index admission (*n* = 7286)	Stay characteristics of all those at first readmission (*n* = 677^a^)	Stay characteristics of all those at second readmission (*n* = 176)	Stay characteristics of all those at third readmission (*n* = 60)	Stay characteristics of all those at fourth readmission (*n* = 31)	Stay characteristics of all those at fifth readmission (*n* = 16)
Average length of stay (in days)	0.739	1.354	2.017	2.783	3.451	2.187
Died this admission (number of people)^b^	NA	0	1	0	0	0
Admission took place on a traditional drinking day?	70.3%	57.9%	48.9%	46.7%	41.9%	25.0%
Average number of days since discharge from last alcohol-related admission	NA	503.8	297.2	239.1	199.1	142.4

^a^ Note that the numbers included each column are not the same as those given in Table Three, because the figures here are cumulative – Table Two notes that 501 people had a first readmission and never came back, but here the total of 677 includes both these people, and also the people who went onto have additional readmissions later

^b^ The “discharge” codes in HES are limited in that they do not capture deaths occurring post discharge from hospital. This can cause incompleteness or bias in some comparative analyses

## Discussion

As a general caution or observation, it should be noted that as a "total population" sample the cohort was relatively large in size (at least in the case of the first regression). Despite this large size, however, the predictor variables achieved significance in the regressions with only modest effect sizes, and only a small amount of variance in readmission rate was explained. An initial conclusion is that alcohol-related readmission is difficult to predict, even with the relatively wide range of variables which were used here.

A second conclusion is that the analysis of multiple readmissions presented here suggests that there is a small group of revolving door problematic alcohol users, even among a young cohort, who start to appear more frequently with alcohol-related problems in hospital, increasingly doing so on non-traditional drinking days, and who stay longer on average once admitted.

The regressions suggest that a number of indicators were associated with readmission. If further research affirms these associations, they give clues about how a young person might be screened steered away from a drinking career at an early stage.

Perhaps the most notable finding, however, and a finding that overlaps with others in the discussion, is the finding that a primary diagnosis of an alcohol-related condition was actually associated with less likelihood of readmission, not more. This variable was included because it was expected that the placement of an alcohol diagnosis as the first code in patient notes (as opposed to the second or later code) was an indicator of how serious their problem was perceived to be by clinical staff, but the regression results suggest the reverse is true. Why could this be? Is it because a clinician labelling these admissions as primarily alcohol-related may encourage some of the patients to seek treatment, thus avoiding their readmission? In support of this idea, a UK trial has shown that patients who personally believed their emergency admissions were related to alcohol were more likely to accept the offer of a alcohol counselling appointment [20].

Possibly this awareness raising could account for some of the correlation with non-return that this variable has, but a more powerful explanation is that the primary diagnosis variable, instead of measuring the seriousness of an alcohol problem, is actually acting as a proxy measure of which cases are relatively "uncomplicated" admissions. Specifically a positive score on this variable is often noting cases in which the three often more notable "complications" of: having a self-harm diagnosis; having another mental health diagnosis; or having another substance use (i.e., non-alcohol) diagnosis do not occur. In support of this, additional analysis of the dataset shows that in 88.5% of the cases where alcohol was the primary diagnosis there was no complicating diagnosis (compared to a figure of 54.1% when alcohol was not the primary diagnosis).

In contrast to some findings [10, 16] but in keeping with others [7, 17], the present analysis showed that a diagnosis of either other comorbid substance use, or comorbid mental health diagnosis are associated with readmission, and the regression also showed the same result for the presence of a comorbid self-harm diagnosis. In addition to the regression results, Table 6 presents some additional breakdowns that show this more clearly - 15.2% of patients with one or more of the three complicating diagnoses returned, compared to 7.2% of those who did not. Therefore given the general lack of overlap between those patients with alcohol as a primary diagnosis, and those with one of the complicating comorbid conditions, it is natural that the former variable has a negative correlation with readmission. The primary diagnosis variable, by unintentionally being a variable indicating how "uncomplicated" an admission is by comorbid diagnosis, illuminates the multiple issues facing some young people with problematic drinking, and also on the effect that these complications have on the continuance of their drinking.

**Table 6:** Return rates for selected categories of patients

Category description	Number in category	Number readmitted	Readmittance rate
Overall	7286	677	9.3%
**Complicating diagnoses** ^**a**^			
Has one or more of the complicating diagnoses	1899	288	15.2%
Has none of the complicating diagnoses	5387	389	7.2%
** Injury**			
Does not have an accompanying injury	6140	604	9.8%
Does have an accompanying injury	1146	73	6.4%
** Injury and complicating diagnoses**			
Doesn't have injury, and has none of the complicating diagnoses	4281	321	7.5%
Has injury, and has none of the complicating diagnoses	1106	68	6.1%
** Gender**			
Female	3733	397	10.6%
Male	3553	280	7.9%
** Gender and complicating diagnoses**			
Female without any of the complicating diagnoses	2529	205	8.1%
Male without any of the complicating diagnoses	2858	184	6.4%
Female with one or more of the complicating diagnoses	1204	192	15.9%
Male with one or more of the complicating diagnoses	695	96	13.8%

^a^ Defined here as having one or more of the following: another (i.e., non-alcohol) comorbid substance use, a comorbid mental health diagnosis, or a comorbid self-harm diagnosis

On the other hand, other admission "complications" were shown not to be associated with readmission. Young people who had a comorbid injury diagnosis were generally less likely to be readmitted, suggesting that these people had less serious alcohol problems, and that many probably only attended hospital primarily because of their physical injuries. Previous research has shown that clinicians are less likely to offer injured intoxicated patients (as opposed to just intoxicated patients) an alcohol referral, and that the injured patients in these situations are less likely to accept a brief intervention if offered [21].

In contrast to previous findings in Germany [7], and Stockholm [10] which suggested that males are more at risk of continued alcohol problems once discharged, young women were found in the present analysis to have more risk of readmission in this analysis than young men. This difference is depicted in Table 6, where young women are shown to have a 10.6% return rate, compared to the rate for young men of 7.9%. This contrast to the previous research can probably be explained in the case of the Stockholm study by the fact that this study included patients from all age ranges. In the case of the German study, the data was collected by way of a follow-up questionnaire in which there was only a 22.7% participation rate, and in which women were significantly more willing to participate. Also in the German study, none of the the alcohol-related outcomes being measured were readmission.

In the present study, again the issue of "complicated" alcohol admission appears to explain much of the higher readmission rate of women. Additional analysis of the dataset shows that young women captured by the study cohort have a generally higher level of one of the three types of complication which have been shown to be associated with readmission (self-harm diagnosis, additional substance use diagnosis, and mental health diagnosis). Young women in the cohort had one of these three complications 32.3% of the time, compared to 19.6% for young men. The index admission characteristics of young men also differed in another major way which helps explain the gender difference in admission rates. Specifically males were much more likely to have injury diagnosis attached to their record than females (27.7% vs 11.2%), and we have seen above that patients who had such a diagnosis were generally less likely to be readmitted. The effect of some of these differences are also reported in Table 6. Both women and men with one of the complicating diagnoses are shown to have elevated rates of readmission, but the return rates for both are much lower when analysis is restricted to "uncomplicated" admissions.

Another variable from the regression relating to patient demographics also predicted readmission, but using it as a criterion for screening would probably be contentious – the average level of wealth of the patient’s area of residence. This is a result in keeping with previous research [22], which found that deprivation predicted alcohol-related admission among their cohort members. Note that there is a possibility that the readmissions rate among young people from wealthy backgrounds would have been be undercounted if they have a non-emergency (but alcohol related) admission into a private facility, but given the near universal use of the NHS in England, this was considered to have only have a slight potential effect on the results.

Although it did not produce a significant predictive effect in the regressions, the variable that measured whether the patient had their index admission on a traditional drinking day did show variation in the sequence analysis. This suggests that as a young person progresses further into a problematic drinking career, they are more likely to have readmissions on days that are not in weekend, not public holidays, and not days that on the eve of a public holiday, a finding which supports existing observations [23, 24]. A variable measuring the ambient temperature on the day members of the cohort were admitted, a variable intended as an additional measure of days of traditional drinking, also did not have a significant predictive effect for readmission.

Finally, the breakdowns in Table 6 give a rough approximation of the broad categories of patients who are most at risk from readmission. Although other results suggest that these categories need to be considered alongside the gender and wealth of a patient, this table shows that the category of easily identifiable patents who are least likely to return are those who arrive with an accompanying injury, and with none of the three named complicating diagnoses (*n* = 1106). This group has a readmission rate of just 6.1%. The next easily identifiable group are those who do not have injury, but who also don't have any of the complicating diagnoses (*n* = 4281), and this group also has a relatively low readmission rate of 7.5%. Finally, however, are the sub-group of young people who do have one or more of the complicating diagnosis (*n* = 1899), and at 15.2%, their return rate is very high. Altogether these three groups comprise the whole of the present cohort (*n* = 7286), and this categorisation may be another useful way of expressing to clinicians the relative risks for different types of young people.

### Methodological considerations

The present findings must be interpreted in the context of several identified limitations.

As has been noted, the regressions only resulted in modest overall findings. More variance overall would probably have been explained if certain other predictor variables had been available. A restriction in a retrospective study of this sort is data availability, and even after using data from outside the HES system (specifically metrological data, and urbanity classifications), some variables could not be included. Specialist alcohol treatment data were not available, and the ethnicity data in the dataset were not usable. The latter issue deprives us of the ability to understand ethnic differences in problematic alcohol use, and hinders health equity. There were also no data on the actual level of alcohol consumption by individual cohort members, and other research (for example, [16, 22]) suggest that this might dwarf the effect of other predictor variables in the present analysis. The usefulness of HES records for research of this type would be greatly improved if they systematically recorded formal screening results for alcohol or substance use, and as recently as late 2015, the need to improve national level alcohol-admission related data has been noted [25].

Second, this study attempts to focus on people at the beginning of a problematic drinking career, but this is not easily operationalized with hospital data which only captures people at specific periods of their life when their drinking behaviour (often briefly) emerges in a clinical setting.

Third, this study is subject to coding variations, omissions, and errors in HES data, as well as mistakenly duplicated records. This problem was partly addressed by pre-processing the data inside the Microsoft Access database application, using a series of proprietary Visual Basic for Applications (VBA) code routines to aggregate and error-check records. These data cleaning routines will not help with other cases, however, for example situations where a person presents with a medical condition that may be attributable to alcohol, but is not noted as such on their HES record in a secondary diagnosis code. This could occur because the young person is not intoxicated upon admission, or because the subjective assessment of a patient’s condition meant that clinicians did not note the presence of acute alcohol intoxication, particularly in cases where blood tests were not performed, or in cases where intoxication was only moderate.

Fourth, the analysis misses out those with such a serious alcohol problem that they die before ever being admitted to a hospital or, specifically with respect to this study, before they are readmitted.

Fifth, linear regression is not an ideal method to use when dichotomous predictor variables outnumber interval or scale type variables [26], as is the situation here. However, no better technique exists, and this was only used for the two subsidiary regressions which, in any case, yielded very little in the way of notable findings.

Finally, people with less serious problems may have been missed. Because the analysis only extended to 3.75 years, it may miss a population of young people of unknown size, with alcohol problems which develop more gradually, who will not be described by this analysis, if they take longer than this to be readmitted. This is a strong possibility that is suggested by the survival curve in Fig. 2, given the probable continuance of the cumulative readmission rate after the end of the 3.75 year period, a continuance that seems likely given the shape of the curve.

Some of the identified problems above are hard to overcome, but the experience of doing this analysis suggests further improvements in any similar research project using HES data or other data of this type. First, an improved study could follow cohort members for a longer time, perhaps for five or ten years. Second, additional variables, such as regional location, a more robust ethnicity measure, and data about actual levels of alcohol consumption by cohort members, would lead to a greatly improved cohort dataset. Some of this data could be gathered via questionnaires at the index admission stage. If gathering data about actual personal consumption is difficult (a likely scenario when research is only being done using routinely collected HES and other data), a possible proxy might be to obtain measures of average alcohol consumption in their residence locality, or to calculate a measure of their local alcohol outlet density (including pubs, supermarkets, and off licences). Third, other types of HES-related NHS data have become available in more recent years, including richer data on outpatient service delivery (including alcohol treatment services) by PCTs, and data on ED activity that does not result in an admission. These fill potential gaps in the analysis here, and would allow a fuller picture of problematic drinking careers among young people. Finally, an additional variable which would be useful in a regression analysis of this sort would be the inclusion of data on NHS expenditure per resident for the alcohol treatment services within the patient's locality.

## Conclusions

The ability of this analysis to predict later readmission was encouraging, and suggests that adding items related to comorbid diagnosis, and perhaps whether their admission was on a tradition or a non-traditional drinking day could contribute to existing procedures designed to screen young problematic drinkers in an ED setting such as, for example, the Paddington Alcohol Test (PAT) [21], or Public Health England’s recommended hospital alcohol pathway for young people [27]. As an incidental side note, it is likely that some in the present cohort were subjected to formal screening (and possibly an intervention) as the use of screening tests by emergency departments increased dramatically over the time of the study period of 2003 to 2008. Only 2.1% of departments used formal screening tools in 2006 [28], a figure that was to rise 51.7% by 2011 [29].

A more refined method of emergency room screening or selective enquiry for alcohol problems would presumably increase the effectiveness and cost-effective of subsequent referral to a brief intervention or other treatment. Using such interventions at weekends and early morning hours could reach the majority of potential cases [30], and might therefore be the most cost-effective approach. Another cost effectiveness implication of these findings is that they can inform calculations by health economists of the extent of personal productivity and socio-economic status loss inherent in a problematic drinking career.

Finally, these findings also show that each geographic area in England will be influenced in its alcohol readmission rates by local demographic characteristics. From the perspective of developing health performance indicators, these results can assist in determining what is a reasonable baseline readmission rate for each area.

## Abbreviations

A&E: Accident and emergency
ED: Emergency department
HadCET: Hadley centre central England temperature
HES: Hospital episode statistics
ICD-10: International classification of diseases-10
IMD: Index of multiple deprivation
NHS: National health service
NICE: National Institute for Health and Care Excellence
PCT: Primary care trust
VBA: Visual basic for applications
VIF: Variance inflation factor
